# Critical Contextual Elements in Facilitating and Achieving Success with a Person-Centred Care Intervention to Support Antipsychotic Deprescribing for Older People in Long-Term Care

**DOI:** 10.1155/2018/7148515

**Published:** 2018-07-08

**Authors:** Lynn Chenoweth, Tiffany Jessop, Fleur Harrison, Monica Cations, Janet Cook, Henry Brodaty

**Affiliations:** ^1^Centre for Healthy Brain Ageing, School of Psychiatry, UNSW Sydney, NSW 2052, Australia; ^2^Dementia Centre for Research Collaboration, School of Psychiatry, UNSW, Sydney, NSW 2052, Australia

## Abstract

Antipsychotic and other tranquilising medicines are prescribed to help care staff manages behaviour in one-quarter of older people living in Australian long-term care homes. While these medicines pose significant health risks, particularly for people with dementia, reliance on their use occurs when staff are not educated to respond to resident behaviour using nonpharmacological approaches. The Halting Antipsychotic use in Long-Term care (HALT) single-arm study was undertaken to address this issue with 139 people 60 years and over with behaviours of concern for staff living in 24 care homes. A train-the-trainer approach delivered person-centred care education and support for 22 HALT (nurse) champions and 135 direct care staff, dementia management education for visiting general practitioners (GP) and pharmacists, use of an individualised deprescribing protocol for residents, and awareness-raising for the resident's family. The HALT champions completed open-ended questionnaires and semistructured interviews to identify the contextual elements they considered most critical to facilitating, educating care staff, and achieving success with the study intervention. They reported that person-centred approaches helped care staff to respond proactively to resident behaviours in the absence of antipsychotic medicines; the champions considered that this required strong managerial support, champion empowerment to lead change, reeducation of care staff, and the cooperation of families and GPs.

## 1. Introduction

Dementia is one of the leading causes of disability in people aged > 65 years. Currently there is no approved treatment available to prevent progression or cure dementia. It is classified in the fifth edition of the Diagnostic and Statistical Manual of Mental Disorders [1] as a major neurocognitive disorder, encompassing a range of degenerative conditions characterised by decline in cognition, impairment in function, and frequently changes in the person's behaviour [2]. As dementia severity increases, certain behavioural responses may change (e.g., aggression declines and apathy increases). Some of the psychological responses in people with dementia include depression, psychosis, aggression, and wandering from home [3]. Diminishing capacity and self-care ability, coupled with increasing psychological responses/behaviours, make it difficult for families to provide the level of care the person requires [2].

In these circumstances, people with dementia require supported care services, including long-term care [4]. One of the main reasons for the use of long-term care in Australians with dementia is the presence of aggression, resistance to care, and extreme agitation, for which antipsychotic and other tranquilising medicines are prescribed in approximately 25% of residents [5]. According to pharmaceutical guidelines, antipsychotic medicines pose high risks for people with dementia and judicious prescribing and deprescribing are recommended [6]. Antipsychotics have been associated with more rapid cognitive decline [7], increased risk for cerebrovascular adverse events (CVAEs, e.g., stroke and transient ischemic episodes), metabolic syndrome, delirium, and death [6, 7]. In view of this adverse event evidence, and the limited efficacy evidence [8], deprescribing antipsychotics is recommended for most people with dementia. When deprescribing is not considered possible because of extreme behaviour, lower doses of antipsychotics are advised alongside nonpharmacological approaches [9].

One of the major reasons that antipsychotic medicines continue to be used in long-term care is that the majority of direct care staff have limited training in dementia care work, and no qualifications in nursing or allied health specialities. They often lack the basic knowledge required for providing quality dementia care and have limited skills in dementia-specific communication and behavioural management [10]. Without exposure to dementia-specific education, these direct care staff are likely to attribute psychological causes to dementia prevalence and the behaviours that they find concerning to a dementia diagnosis [11]. Consequently, while this staff is charged with providing care and support for people with dementia, often with very limited supervision from a qualified nurse, they may find it difficult and burdensome to manage coexisting physical, behavioural, and mental health issues [12]. Direct care staff may, therefore, struggle to communicate and engage the person with dementia in meaningful conversation and leisure activities and may not know how to prevent and deal with resistance during a care event [12].

As well, without dementia-specific knowledge direct care staff have less hopeful attitudes towards people with dementia, do not anticipate meaningful responses from them, and have low expectations of improvements in the person's functional and cognitive ability [13]. In these circumstances, staff may disengage from the person psychologically and socially and can inadvertently reinforce the behaviour they find challenging by reacting negatively to the person and/or the behaviour [9, 13]. Staff may also fail to support the person's positive, prosocial behaviour when they are verbally and physically agitated [12]. Targeted education can help to address negative staff behaviour by clarifying misconceptions about cognitive impairment in dementia, give advice on the different reasons and remedies for the person's behaviour, and increase staff's confidence and skills in providing individualised care [14].

Dementia education based on a person-centred care framework [15] helps long-term care staff to recognise that many of the person's behaviours, such as agitation and resistance to care, arise from the person's interactions within the care context. Through exposure to this educational focus, staff come to understand that people with dementia, like themselves, exist in a social, relational context, and that when staff make genuine attempts to develop positive and enriching interpersonal relationships with people with dementia, they can help to reduce the disabling effects of dementia and promote well-being [16]. A person-centred approach to dementia care places considerable value on understanding the person's unique identity and personality, helping the person to feel a sense of self-worth and value through tailoring services to the individual's psychosocial needs, providing options for continued social engagement and access to meaningful life activities, and including the person in decisions about their care and daily life experiences as much as possible. This approach to dementia care is effective in improving the quality of care, such as bathing and social and recreation activities [17, 18], improving well-being [19], reducing various forms of agitation [20, 21], and reducing the need for antipsychotic medicines to manage behaviour [21].

For person-centred care to become a practical reality in reducing, or eliminating, the use of psychotropic medicines in people with dementia, its application must be interpreted in the context of the person and their personal characteristics, collegiate relationships, and the healthcare organisational circumstances [16]. The model is best implemented when the entire system is framed by policies to guide implementation across the organisation, as well as within supportive medical and ancillary services [16]. Leadership at senior and middle management level is a key factor in effective, system-wide adoption of the person-centred approach [15, 16], including willingness to introduce targeted education, training and supervision for all direct care, and support and ancillary staff [22]. Despite the evidence for a person-centred approach, many long-term residents with behavioural issues are still prescribed antipsychotic medicines [5].

In response to the continued reliance on antipsychotic medicines for people with behaviours that cause difficulties for long-term care staff, we conducted the Halting Antipsychotic use in Long-term care (HALT) study [23]. The study implemented a multifaceted intervention, using a train-the-trainer approach to deliver person-centred dementia care education and support for HALT (nurse) champions and direct care staff, dementia management education for visiting general practitioners (GP) and pharmacists, awareness-raising for the resident's family and use of an individualised deprescribing protocol for participating residents which followed Australian guidelines stipulating a dose reduction of 50% every 2 weeks and ceasing after 2 weeks on the minimum dose, withdrawing one antipsychotic at a time, with risperidone (if prescribed) to be withdrawn last [24]. Some of the participating GPs prematurely applied the deprescribing protocol for 38 of the residents. The GPs' adherence to the deprescribing protocol is the subject of a HALT substudy, with a manuscript in process. While 133 (94.7%) of the participating residents were initially deprescribed, there was a cessation or a reduction in dose of antipsychotic use in four out of five of 93 residents remaining at 12-months follow-up, without an increase in substitute medicines. The number of participants on regular antipsychotics over 12-months reduced by 81.7% (95% CI: 72.4–89.0) [23].

This HALT substudy aimed to identify the champions' experiences with applying the person-centred care training when providing leadership to direct care staff in person-centred dementia care. In this article we focus on the champions' use and satisfaction with the training program resources and recommended techniques, their perceptions of the enablers and barriers to person-centred approaches in practice and whether the person-centred care approach helped to reduce resident behaviours, and their reflections on how the person-centred care approach adopted had an influence on the culture of dementia care.

## 2. Materials and Methods

The HALT study**, including this substudy,** was approved by the university's research ethics committee (HC13203) and was registered with the Australian New Zealand Clinical Trials Registry (#12614000309684), with study methods and results reported according to the COREQ guidelines.

### 2.1. Setting and Participants

HALT was conducted as a single-arm longitudinal study in a convenience sample of 24 long-term care homes (12 private, 10 charitable, and 1 community-based, each with >60 beds) in the greater Sydney region, Australia. Informed proxy consent was obtained for all but one of the study participants; one resident was able to provide her own consent. Appointed nurses from each of the homes recruited 149 people aged 60 or older, who lived in the home for ≥1 month and were taking regular antipsychotic medicines for ≥3 months, of whom 139 were eligible. Eligibility was assessed via staff interviews and file audits. Residents were excluded if terminal illness or a primary psychotic condition was present, or if they had extreme behaviour that might be too difficult to manage in the long-term care setting (score ≥50 on the nursing home version of the Neuropsychiatric Inventory (NPI-NH) [25], domain scores of 12 for at least two of delusions, hallucinations, agitation/aggression, anxiety or disinhibition, and occupational disruptiveness score ≥4 for at least two of these domains). The extreme behaviour exclusion criterion was informed by contemporaneous Australian Department of Health rules for severe behaviour supplement [26].

The 22 HALT champions recruited to the study were volunteer registered nurses from each of the participating homes who were identified by their managers as having leadership qualities in educating/training and supervising direct care staff in person-centred care techniques for participating residents. The study's project coordinator recruited and consented one or two experienced senior nurses within each of the 24 participating care homes to take on the champion role.

### 2.2. Study Intervention: Person-Centred Education/Training and Support

The person-centred education/training and support component of the multifaceted study intervention used an evidence-based train-the-trainer approach to deliver person-centred dementia care education and support** [16, 19]** for HALT (nurse) champions and direct care staff. Once consented the champions attended a 3-day workshop delivered in groups of 5-8 by the study dementia nurse specialist. Champions were provided with a training manual and set of dementia resources which outlined practical strategies for problem-solving issues with individual resident behaviours. The main learning objectives of the champion education/training course included educating direct care staff to value and respect the resident in all interactions; regard the resident as a complete individual; seek to understand the experiences and behaviour of the resident from their perspective; understand that all resident behaviour is a form of communication; and make every attempt to create a positive care and social environment to prevent the development of agitated behaviour.

Experiential and adult learning techniques were used in the training program, including role play of person-centred approaches with champion-identified examples of known residents with behaviour that was concerning for direct care staff; trainer demonstration of how these behaviours might be investigated and approached during role play; group activities which encouraged expression of feelings through sharing observations, and engaging with emotions by entering into the reality of the person with dementia and not simply trying to distract them; and facilitating shared learning by assisting the champions to use the different learning resources and techniques in preparation for their role, such as care planning with reference to residents recruited to the study, and use of practice memos and mini-tutorials on person-centred approaches that they planned to use during staff handovers.

Along with a range of strategies to facilitate person-centred care practice with direct care staff, the champions were provided with helpful resources such as person-centred care plans which focused on maintaining resident function, engagement, and well-being; care management and behaviour prevention/reduction techniques and protocols; case management and team leadership techniques; the VIPS[16] model of person-centred care; relationship-building processes; and transition and continuity planning. The study's emergency behaviour response protocol (Supplemental File (available here)) included these key person-centred approaches when investigating, interpreting, and responding to resident behaviour that staff found extremely challenging.

Champion education and supervision of 135 direct care staff occurred in consultation with their managers, the project coordinator, and the dementia nurse specialist, which continued on a regular basis until follow-up. The dementia nurse specialist and the project manager provided on-site and phone support to the champions as they commenced educating and supervising direct care staff in person-centred care and behaviour management.

### 2.3. Measurement and Data Collection

The HALT substudy identified the contextual elements that the champions considered most critical in facilitating, adhering to and achieving success with the person-centred care component of the HALT intervention, and how this change process impacted on care delivery and outcomes for participating residents from the champions' perspectives. To obtain these data a questionnaire was completed by HALT champions before and after they participated in the education/training program, and they were interviewed at study follow-up.

The pre/post education/training questionnaire was based on process evaluation principles and included eight opened-ended questions on the champion's learning needs, expectations, and achievements, including their knowledge of dementia and dementia care, application of dementia care skills/strategies, and knowledge and application of person-centred responses to behaviour in dementia. One additional item was included in the posteducation course questionnaire which asked the participants to rate the program quality on a Likert scale ranging from 1 ‘did not meet learning needs' to 4 ‘met all learning needs'. Questionnaires were administered by the research assistants immediately prior to and at the conclusion of the champion's education/training course, and participant responses were handwritten. Completed questionnaires were returned to the study research assistants in sealed envelopes for data entry and analyses.

One-on-one champion interviews were informed by the substudy aims. The interviews were conducted by the project coordinator in private rooms within the aged care home, using a semistructured interview guide. For a small number of champions who were unable to be interviewed during working hours, one-on-one interviews were conducted by telephone. Interview questions included a mix of closed and open-ended items, and items which asked participants to give ratings to fixed questions using Likert scale responses, such as ‘none', ‘a little', ‘some', ‘a lot' and ‘substantial' and ‘very low', ‘low', ‘neutral', ‘high', and ‘very high'. The 10 topics covered included work-related demographics; progress and details on instituting person-centred approaches in care for included residents; barriers and enablers with instituting the person-centred approach; strategies used successfully in reducing triggers and resident behaviours; impact of person-centred approaches on resident behaviour; impact of on-site person-centred education, training, and supervision on care practices; and factors involved in instituting a person-centred workplace culture. Champion responses were tape recorded and key statements were hand-recorded on the survey and interview forms. Handwritten statements were checked for accuracy with participants at the end of the survey. Since the tape recordings were transcribed verbatim, they were not returned to participants for their review.

### 2.4. Data Analyses

Questionnaire responses, interview transcripts, and notes recorded by the data collector were sorted and classified with computer software, NVivo 8[27], and were content analysed by experienced qualitative researchers. Three study team members with expertise in this form of data analysis independently employed an iterative process to code these data and identified the core concepts, deriving key themes from the data as a whole. Thematic analysis was guided by the* a priori* topic areas of the champion questionnaire and semistructured interview questions. Emphasis was placed on subjective experiences and the meanings that the champions attached to that experience [28]. Key themes were independently analysed by two of study team members for champion questionnaire and interview responses and then confirmed and/or corrected by the third team member [29]. Consensus was reached between the three study team members regarding the categories and themes for each of the key domains of interest. This approach to data analysis produced an in-depth interpretation of the dynamic and subtle interplay of factors occurring at the individual, group, and organisational level in regard to the implementation of the study intervention, its potential generalisability, and optimisation in routine long-term care practice [30].

## 3. Results and Discussion

### 3.1. Results

The substudy results are presented according to the questions which guided the champion questionnaires and interviews, concluding with the themes derived from these data.

#### 3.1.1. Work-Related Demographics

The 22 champions were all female registered nurses, with a range of educational qualifications and working at different levels of authority. Educational preparation included hospital-training certificate and/or diploma and/or a bachelor and/or a master degree in a health discipline. Champion work roles included senior registered nurse (n=6), clinical nurse specialist (n=4), clinical nurse consultant (n=5), nurse practitioner (n=1), quality manager (n=3), deputy director of nursing (n=1), and care unit manager (n=2). Most of the champions had participated previously in some form of dementia-specific education within the care home or externally, ranging from certificate to degree-level courses.

#### 3.1.2. Achievement of Learning Objectives

All 22 champions responded that the education/training course had met many of their learning objectives, particularly on how to use a person-centred care approach to reduce the incidence and severity of resident behaviour, techniques in communicating with residents with dementia and behavioural issues, and how to change staff's reliance on antipsychotic medicines for resident behaviours they found difficult to manage. They considered the course had taught them strategies which they intended to utilise in practice as a way of facilitating implementation of person-centred care. The following statement of one champion's satisfaction with the education and support received was similar to sentiments expressed by most Champions:

“Staff have received excellent education and feel confident and involved in the project. They have found that the process has been very consultative” (SC02).

The most useful strategies included how to interpret a need from behaviour, procedures for managing complex change, showing direct care staff how to be therapeutic agents, how to use practice improvement memos, microtraining and person-centred care planning, and how to use the VIPS [16] model in emulating the person-centred care approach in practice. The postcourse questionnaire responses indicated that champions considered there was a need to know more about psychotropic medicines and how to manage resident aggression, especially in care delivery. A common response was the need for dementia specialists or educators to be available for onsite follow-up support or training for direct care staff. On a scale of 1 ‘did not meet learning needs' to 4 ‘met all learning needs', 10 champions gave a rating of ‘3' and 12 gave a rating of ‘4'.

#### 3.1.3. Progress with Instituting Person-Centred Approaches in Care

All 22 champions were positive about the HALT program before and after its introduction, and this sentiment continued at follow-up. As one of the champion's reflected:

“I think the project has created awareness that anti- psychotic medications are dangerous and not always the answer. It has allowed staff to witness first-hand the behaviours of residents who have been successfully de-prescribed. It has shown that it is a myth that behaviours automatically increase when antipsychotic medications are decreased” (MC01)

The champions reported that once they introduced the strategies they learned through the education/training course within the care home, the majority of the direct care staff were enthusiastic about the person-centred care approach and were willing to apply these approaches for behaviour that was troubling for the resident and themselves. Direct care staff were also curious to see whether person-centred approaches to behaviour prevention and management were useful. Most of the champions found that direct care staff were willing “…to give it a try and see how it goes” (VC01).

It was universally agreed that the managers provided good support for the champions and enabled them to undertake the level of education, training, and support they needed to pass on the learning obtained during the education/training course. Time was set aside for champions to work directly with staff. Shared governance with champions was a feature of the managerial leadership for the project, evidenced by the initiatives taken by champions to educate staff in person-centred approaches to care, and in the learning strategies they used for person-centred responses to resident behaviour. However, 18 out of 22 champions advised that they undertook a high level of unpaid out-of-hours work to initiate and continue the education, training support for staff. In a few cases the managers rostered champions off-duty, but paid them, to provide staff education/ training and support.

For the most part champions were fully involved in implementing the onsite education/training using many of the learning resources provided, including person-centred care and lifestyle plans, mini-tutorial and case conference protocols, practice memos, role play guidelines, and behaviour response flow charts. They took a range of opportunities to educate, train, and support staff colleagues, including during shift handover times, in targeted education sessions with care staff, in regular debriefing sessions to review progress with targeted staff, and conducted case study reviews with the whole team, case conferences with the family, nurses and allied health staff, and supervision of staff in daily practice. These sessions ranged from “*daily mini learning tutorials at shift handovers*” (MRC02), “*20 to 30-minute education sessions with direct care staff up to four times each week*” (SBC01) and “*education sessions of one or more hours once each week*” (SLC01), and some champions “*gave advice during case conferences on specific non-pharmacological techniques suitable for individual residents*” (BHC01).

The champions attempted to educate, train, and support as many staff as possible, estimating that they reached approximately 75-80% of all staff, including staff working on weekends in some of the homes. Education techniques were based on the HALT person-centred care training program folder and resources, supplemented by education provided by external consultants and via DVDs and other online resources provided by dementia support and training services. All 22 champions considered that staff's level of knowledge and awareness of nonpharmacological management of resident behaviour improved with the education, training, and support they received through the HALT intervention. They recounted different ways that the techniques taught in the course helped them to understand and work on minimising triggers which individual residents found distressing. One of the champions explained this process:

“We checked the reasons for agitation, such as constipation, pain and thirst, and attended to any issues that seemed to be a trigger. We also asked the doctor to attend to issues quickly, such as pain” (SLC01).

While the support from the study dementia nurse specialist and project coordinator was appreciated by champions, it was generally thought that more onsite and on-going education was needed for all staff. Two of the champions requested that “*HALT staff explain the concepts to all staff, on all shifts*” (BPC01),* “as some staff need to have simplified a version of how conform with the care protocol*” (SC02). Where the champions were not involved from the beginning of the project, such as when new champions were enrolled following the resignation or transfer of a former champion and/or care unit manager, there was a higher need for onsite education and support by the study team members. As direct care staff turnover continued to be an issue in some of the homes, the champions found it difficult to ensure all new staff were exposed to the same level of education and support in person-centred approaches to care as occurred with more stable staff. It was acknowledged that “*continued, on-site education and training in nonpharmacological management of resident behaviour*” (BHC02), with the support of managers, was essential to continue deprescribing of psychotropic medicines for current and future residents with dementia.

An issue raised by six of the champions was the need for additional onsite education of person-centred approaches for direct care staff from culturally and linguistically diverse backgrounds (CALD), where more discussion and experiential education for understanding these concepts and their practical application were required. Where a facility had a high proportion of CALD families and residents, it was suggested that translators and written translations would assist in the implementation and acceptance of the person-centred approach. This issue was explained by one of the champions:

“These staff need to first of all be helped to understand the concept of helping residents to make decisions for themselves and to express their own personality, then feel confident to try different approaches to meet the resident's needs, because all residents are different, and they have different issues and respond differently to triggers that support their well-being and also ill-being. If the information about the care model could be translated for the overseas-born staff that would help” (BPC02).

#### 3.1.4. Enablers and Barriers in Adhering to the Person-Centred Approach to Care

The context of the care environment proved to have the greatest impact on the operationalisation of person-centred care practices. Champions reported that their work cultures were developed to value, respect, empower, and give choice for the champions, the staff, and the residents. The organisational climate and culture created by the managers supported the committed vision of a person-centred approach to behaviour reduction and management and also influenced the actions and interactions of the staff providing the care. The champions spoke of how direct care staff were encouraged and assisted to put their relationship with the person before the tasks they needed to undertake. This was greatly helped by champions working with staff to plan care and behaviour management in ways that focused on the person's unique preferences and needs. The perceived enablers and barriers to making these practice changes are listed in Table 1.

#### 3.1.5. Staff Progress in Using Person-Centred Responses to Resident Behaviours

The 22 champions considered that the majority of direct care staff derived deep satisfaction and meaning from their work and the relationships that they formed with the residents, particularly after learning how dementia affects the resident's communication and other abilities. They advised that most of the direct care staff worked together to provide a sensitive way of understanding and responding to each resident's needs and unique personality. Champions identified that this approach helped to provide culturally appropriate solutions in regard to behaviour management, “*because it was based on the unique priorities and perceptions of the person, their family and their cultural context*” (SLC01).

An important change for direct care staff was the incentive for them to minimise, and challenge systems-driven services that were not in the best interests of the resident. This was enabled by managerial and champion leadership in shifting in the organisation's control over care schedules, and their role in negotiating schedules and care/treatment priorities with families and GPs. This leadership created a climate throughout the care home and allowed the majority of champions sufficient time and resources to lead the change process.

The champions reported seeing improvements in the quality of care for residents with facilitated learning in practice. Words used to describe these improvements included staff ‘*being more responsive'* (SLC01), “*more respectful*” (SBC02), “*giving care in a timely way*” (VC01), and ‘*being more confident in responding to difficult behaviours*” (BHCO2). Knowledge and skill transfer were reported to occur across the staff group as a whole, with emphasis placed on discussing behavioural issues and management for different residents at staff meetings, case conferences, and shift handovers. Staff became more proactive in requesting that residents' GPs deprescribe antipsychotics and prescribe regular analgesia where the staff identified this as a potential, or actual, cause of agitation and resistance in care. The most commonly stated improvements arising from the education and training were staff's recognition and removal of behaviour triggers relevant to the individual resident, and the staff's identification and responses to the residents' unique needs.

It was noted by nine of the champions, however, that some direct care “*staff needed much more education and training, as well as role modelling and practice supervision, than others” (WC01), since “if the resident is not settled, no matter what you try, the staff do not know where to go with this*” (BHC01). For these staff the most successful learning came through witnessing successful responses to resident behaviours by their colleagues and the champion, and by having opportunities to practice these techniques through role modelling. Once successes in reducing distressing behaviours were observed and/or experienced by direct care staff and then discussed among the team, these staff gained more confidence to initiate new approaches to care.

#### 3.1.6. Strategies Used Successfully to Reduce Triggers for Resident Behaviours

The major strategies that all champions found useful when helping direct care staff to become aware of potential triggers for individual residents included searching out information on the resident's previous lifestyle and history from family and client records, discussing the resident's unique needs with nursing care and allied health teams in dedicated meetings and at case conferences, and spending more time with the resident to learn about their preferences in care. As one champion noted:

“… taking small, well-planned steps (towards change) helps to convince the staff of how important it is to take notice of what the resident is trying to tell us” (SBC01).

Another champion identified that

“…. helping one resident (to be less agitated), helps other resident to feel settled” (VC01).

A frequent example given of the type of change made by direct care staff included paying attention to the potential for residents to experience pain, which resulted in “*better pain assessment and an increase in needed analgesia*” (BHC01). It was considered by all 22 champions that regular pain relief greatly reduced resident distress, even when aggressive and agitated behaviour was severe. Other pain/discomfort-relieving methods were also reported, such as having “*the physiotherapist ensure that the resident had correctly fitting shoes and was involved in a more frequent exercise program*” (MRC01).

A further strategy reported by 17 of the champions in reducing behaviour triggers was allocating time for direct care to communicate with residents when verbal agitation was present, and where this appeared to be “*associated with loneliness and boredom*” (BLC01). As well, 20 of 22 champions reported that they facilitated one-on-one communication responses and individualised care for residents during periods of extreme agitation and other forms of distress. These changed approaches to behaviour reduction and management helped direct care staff to realise that resident behaviour was often associated with an unmet physical and/or psychosocial need. Direct care staff were, therefore, “*more interested to discuss and collaborate on solutions to reduce triggers for resident behaviours*” (WC02).

#### 3.1.7. Perceived Impact of the Person-Centred Approach on Resident Behaviour

Since all 22 champions facilitated person-centred care with direct care staff, they recounted numerous examples of their observed changes to care practices and the impact these changes had on participating residents. All champions considered that the person-centred approach was helpful for the majority of residents, not just those enrolled in the study. Reported improvements in outcomes for participating residents were described in terms of functional status, overall well-being, and behaviour reduction. Improvements reported included the residents being calmer, more cooperative, more social, more independent in activities of living, and less anxious. The champions reported that these positive changes tended to occur gradually at first, but “*once the staff became aware of how their care and therapy practices had an influence on the resident's function and well-being, staff made greater efforts to identify and meet individual resident *needs” (SLC01). With regard to observed improvements in resident function and well-being, the champions noted that individual residents increased their involvement in communicating and engaging with others in pleasurable activities, their appetite and sleep time and quality improved, and the incidence and severity of agitation reduced, such as “*repetitive calling out*” (OCC01), “*screaming*” (BPC01), and “*resistance to personal* care” (RSKC01). Some champions observed that the residents who were responsive to person-centred care approaches were* “more alert, were able to mobilise more frequently and had less falls”* (SBC02).

Nevertheless, some resident behaviours and activities of living were not observed to improve with deprescribing. Two of the champions noted that these residents were “*mostly women with nonaggressive behaviour*” (VC01) and “*with no prior mental illness*” (SLC01). Where residents had mild-moderate behavioural issues prior to deprescribing, the champions observed minimal change in these behaviours. Two of the champions advised that where deprescribing produced no changes in resident function, well-being and agitation, this may have been related to the resident selection, i.e., “*residents with less troubling behaviours*” (WC01). It was noted, however, that minimal reductions in behaviour incidence and severity was likely a positive outcome for these residents, given the proven iatrogenic adverse effects of psychotropics in dementia [7]. As previously identified, two of the champions reported that a small number of residents had a return of previous behaviours associated with long-standing mental illness

#### 3.1.8. Factors Involved in Instituting a Person-Centred Workplace Culture

Champions recounted many examples of how their care home managers, champions, and direct care staff developed a coordinated system of sharing the common values of person-centred dementia care, while understanding the value that their unique roles played in caring for residents. Improved communication systems between 19 of the 22 champions, managers, and direct care staff also helped to share knowledge about residents, in order to develop greater awareness of what precipitated and helped to reduce agitation and other behaviours in residents. The champions recalled how positive interactions between direct care and therapy staff, and managers, were key to supporting the personhood of individual residents; this occurred mainly by developing empathetic working relationships.

The other important approach to developing a person-centred culture occurred through the champions working hard to establish care partnerships, through enabling direct care staff, residents, and families to have direct involvement in decision-making in care schedules and treatment regimens. Champions spoke of how partnerships were formed with families through staff developing an understanding that each resident had a unique history and that residents and/or families were entitled to indicate their preferences in daily living activities and the way that care was provided. As one champion found:

“Helping the family to get involved in discussing their relative's issues and needs, and what might be causing their distress, this will help the staff to feel more connected and more willing to work with the family to sort out these issues.” (AHC01).

By taking this approach, direct care staff learned how to humanise care practices, how to help residents maintain their personhood, dignity, and decision-making; and how to individualise care and therapy activities that were preferred by the resident and/or their family, because “*each person is different and has different issues, so different approaches are needed to help them*” (BHC02). Staff learned to recognise the centrality of creating an environment for positive relationship-building between themselves, the resident, the families, and each other in assisting the resident to be less stressed in day to day living.

Champion recommendations for further development of a person-centred workplace culture included: “*education and training for all staff in person-centred care approaches, as well as in how to recognise and reduce behaviour triggers*” (OOC01); “*establishing mechanisms in care schedules/regimens to allow direct care staff to get to know their residents' backgrounds, personalities, preferences and needs*” (WC02); “*welcoming and encouraging family involvement in discussions of residents needs and preferences*” (BLC01); “*providing meaningful daily living activities in ways that create well-being for the resident*” (WHC01); “*personalizing the care environment in ways that help the resident to feel safe and calm*” (RKC01); “*enabling direct care staff to offer flexible care routines without repercussions from other departments such as catering*” (BPC01); and “*providing managerial leadership for staff continuity and flexibility in care delivery*” (WMC01).

## 4. Discussion

This substudy of the HALT trial provided data on the champion's experiences of how person-centred training program helped them to provide the groundwork for a change in dementia care, their observations of the impact it had on resident behaviour, and their perceptions of its influence on dementia care culture in relation to the underlying mechanisms of the care context, management systems, staffing arrangements, and resident issues. While this substudy does not report on the answers to these questions from the perspective of the study's GPs, families, direct care staff, and care managers, the champions' insights provide valuable guidance on how staff leaders can progress needed change, and in identifying what structural factors play their part in successful implementation of person-centred care. The main themes arising from these data focus on organisational and staff readiness to accept, believe in and embrace practice change, by instituting a planned change management procedure, empowering champions to drive the change process, and empowering direct care staff to adopt the change in care practices. The champion interview findings indicate that success with implementation related to four key factors: behaviour management improvement was considered a priority for champions and managers; leadership was in place to support person-centred care across the care unit; a ‘bottom-up' change model worked alongside ‘top-down' change approach; and the person-centred approach to behavioural issues was appropriate to the context.

These findings contribute to the literature by explaining the discrepancies between the expected and observed outcomes for some residents, help in understanding how the care context influenced outcomes, and provide insights to aid future implementation of person-centred care for residents with behaviours that are concerning for direct care staff [31]. The champions also provided many useful suggestions on the potential generalisability of the change processes employed and optimisation of person-centred care in routine long-term care practice, similar to recommendations arising from previous person-centred care research [31, 32].

The positive outcomes reported by the champions were dependent on their acquisition of detailed knowledge, skills, confidence, and competencies for leading the change process. It appears that many of the different resources and strategies gained through the training course, such as practice memos, mini-tutorials, person-centred care plans, and the VIPS [16] approach to implementing person-centred care, were helpful in applying knowledge into practice. As identified in a previous person-centred care study, the onsite and telephone follow-up support provided by the project coordinator and the dementia nurse specialist helped the champions to consolidate their learning and to improve their confidence in facilitating learning in practice [32].

Nevertheless, as four of the champions reported finding it more difficult to institute required changes in care practices, despite managerial support and authority delegation, the shift from knowledge gain to knowledge translation may be related to the characteristics of the individual. This finding indicates that one-off education/training courses were insufficient to arm even senior nurses with the knowledge and skills they needed for initiating and providing leadership in practice change [31, 32]. As advised by these champions, additional onsite mentoring and support by the study's dementia nurse specialist would have been very helpful in boosting their confidence in applying the strategies gained through the education/training course.

Similar to other studies of the person-centred care model in long-term dementia care, managerial leadership and cooperation, direct care staff understanding, knowledge, acceptance, and perseverance in applying these approaches, as well as GP and family acceptance, were considered essential to implementation success [20, 31]. At times, champion leadership for the study intervention was an issue for those from a culturally and linguistically diverse (CALD) background who had difficulties with challenging the ambivalence of some of the residents' GPs and families with respect to the benefit of deprescribing antipsychotic medicines and replacing such treatment with person-centred behavioural management techniques. The authority provided to the champions by senior management was identified by the champions to be a key factor in addressing the reluctance of some families and GPs to accept and support a nonpharmacological approach to behaviour management. Consequently, the family's and GP's understanding and acceptance of the person-centred response to resident behaviours were an important factor in its implementation at the individual resident level. Enabling the person-centred approach at the organisational level in this substudy required dedicated education and skill training for staff who felt insufficiently empowered to question medical decisions [15, 16]. In these circumstances, the champions found it useful to engage the services of the study's psychogeriatrician to influence the medical approach to dementia in long-term care [23, 33].

The study findings support the contention that person-centred improvements in long-term care services are largely a function of organisational structure, workforce capabilities, organisational climate, communication structures, staff readiness, and leadership [16, 19, 32, 34, 35]. One of the strengths of the HALT study was the high level of organisational support to enable champions and direct care staff to implement and sustain person-centred care practices. Examples of how managers supported the change process included modifying work schedules, resident care priorities and resident care plans, clear communication procedures among the work teams, dedicating time for staff education and supervision in practice, providing different practice opportunities in relation to behaviour management, and supporting adjusted treatments for participating residents. As posited by Brooker [16], concurrent consultation streams of activity in knowledge translation need to occur with development of person-centred responses to behaviour. This concurrent process required active engagement of all stakeholders, including resident families, GPs, and direct care staff, in interpreting and translating the findings to practice improvement opportunities [36]. Such intensive activities demanded additional work for the champions and a redefinition of their work roles and rescheduling of their time. It also impacted on the job requirements and accountability of the direct care staff. These requirements are often a challenge with practice change at the organisational level [31, 37].

While the study findings are most informative in reporting details on how to introduce and sustain person-centred approaches to behaviour management in long-term care, they are limited to the experiences and recommendations of the study champions who provided onsite leadership and facilitated the approach with direct care staff. An acknowledged limitation is that the champion interviews were undertaken by the project coordinator, which may have introduced bias in the champions' responses. Nevertheless, these data provide detailed reflections on the factors the champions observed as having influenced resident outcomes with a person-centred approach and enhance the HALT study's contribution to the nonpharmacological management of behaviour in dementia [8, 18, 23, 33]. Future studies on how to best gain acceptance of this approach by resident families and GPs are recommended. As well, a more comprehensive view of the associated change practice factors and effects on resident outcomes from the perspective residents' families and GPs, direct care staff, and care managers would have been informative. While this is one of the substudy's limitations, these data are the subject of other HALT substudies and future publications.

## 5. Conclusions

There is strong evidence in the literature to suggest that person-centred responses can help reduce behaviours in long-term care residents which care staff find challenging, in respect of being able to provide them with personal care without resistance and in helping the person attain well-being, despite physical, social, and cognitive limitations [16–20]. These findings are similar to the experiences of the HALT champions and are confirmed by the HALT study results, which showed that at 12 months follow-up approximately three-quarters of the HALT study residents remained deprescribed and showed no changes in behaviour [23]. Translating the person-centred knowledge and skills learned in the champions' training course required a whole systems approach to initiate and sustain care practice changes. This was enabled by strong managerial support for the person-centred approach to behavioural responses, empowerment of the champions to drive and facilitate change at the site level, and understanding, acceptance, and engagement with the person-centred approach by direct care staff. Adherence to the HALT deprescribing protocol [23] and acceptance of the person-centred responses to resident to behaviour also relied on the willing cooperation of residents' families and GPs. Their cooperation provided nurses and care staff with opportunities to adjust organisational systems and care practices to accommodate unique resident needs and preferences, thereby more readily addressing issues that triggered behaviours when providing care. Empowering champions to facilitate onsite staff education and supervision presented care staff with greater opportunities to reflect on and adjust care practices and communication strategies that achieved better outcomes for residents. Further research is now needed to collaborate with key stakeholders on how best to promote the benefits of person-centred care as a viable alternative to antipsychotic medicines for long-term care residents.

## Figures and Tables

**Table 1:** Enablers and barriers to implementing person-centred care approaches.

**Enablers for the person-centred care approach**	

Enablers	Examples provided by Champions

Management support for incorporating personalized care into daily care practices	“…*allowing residents to sleep-in and providing them with breakfast when they awoke and requested it*” (SCC01)

Management allowing Champions and staff to try out new ideas	“ *…. letting the nurses try different approaches for some residents….and discovering what worked, and what was not working*” (SLC01)

Shared governance in decision-making	“… *input and feedback and discussion with direct care staff on resident preferences, needs and issues*” (WMC01)

Clear communication between managers, nurses, direct care staff, the Champions, the resident's family and the GPs	‘… *good communication greatly improved the buy-in by all stakeholders and the capacity for speedy problem solving on issues regarding resident health and well-being*” (AHC01 )

**Barriers to the person-centred care approach**	

Barriers	Examples provided by Champions

Reluctance of residents' families to agree to deprescribing	*“....it was important for Champions to give families and GPs advice on the person-centred approach to behaviour reduction and management, to show how this approach was embedded in the approved deprescribing protocol and to emphasise that the approach was unlikely to cause any harm to the resident”* (MC01)

Reluctance of resident's GP to deprescribe	*“… the GPs needed to feel they were making the decision based on knowledge and evidence*” (BHC01)

Reluctance of some nurses to support deprescribing	“*some RNS would only agree to comply if the doctors allowed it*” (BPC02) *“ some direct care staff expressed fear to RNs (nurses) that they could suffer assault from a physically stronger and aggressive resident*” (SC02)

Negative family attitudes towards non-pharmacological management of behaviour	“*a culture of blame seemed prevalent if staff were unable to contain the resident's behaviour,*” (WDC01)

Task-focused care culture	*“…. not having the person-centred approach in the forefront of their thinking amid all the things they have to do for residents*” (BLC01)

Time to implement person-centred behaviour responses	‘…(direct care staff) *being too busy to really take notice of what was going on for residents*” (WC01)

Lag time in reporting of the study findings	“….(Champions) *having insufficient feedback on the study's progress*” (MRCO2), *“… (study findings) would have helped to inspire the direct care staff to maintain their interest and commitment to person-centred approaches to behaviour reduction and management*” (BHC02)

## Data Availability

Some of the data are currently still being analysed for other studies and will not be available until all articles are published.

## Supplementary Materials

Supplementary Materials The HALT emergency behaviour response protocol was used by the study's nurses and champions when investigating, interpreting, and responding to resident behaviour that staff found extremely challenging, including person-centred approaches.
