# Supplementary material for: Critical Contextual Elements in Facilitating and Achieving Success with a Person-Centred Care Intervention to Support Antipsychotic Deprescribing for Older People in Long-Term Care
Source: Biomed Res Int. 2018 Jul 8;2018:7148515. doi: 10.1155/2018/7148515 (PMC6057399; doi:10.1155/2018/7148515)
Supplement: Supplementary Materials — The HALT emergency behaviour response protocol was used by the study's nurses and champions when investigating, interpreting, and responding to resident behaviour that staff found extremely challenging, including person-centred approaches. [file 7148515.f1.pdf]

Emergency Protocol – HALT

In the event that a HALT participant experiences an acute behavioural disturbance, please follow the protocol outlined below.

1. Assess the person and their environment and minimise any immediate risks to the safety of the resident, staff and others in the area

2. Attempt to narrow down possible causes of the current episode of BPSD[#]

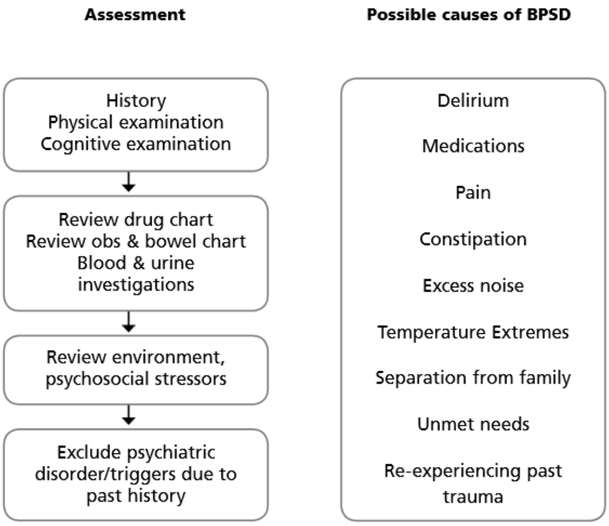

3. Refer to the resident's care plan and try one or more of the behaviour management strategies recorded in the resident's file. Note how the resident responds to the intervention and re-assess the situation.

4. Apart from trying to engage the person, consider the following non-pharmacological actions[#]:
   - Prevent self-harm, such as keeping the person away from open windows, balconies, electric cords and other equipment.
   - Remove from the immediate environment others who may be at risk.
   - Move the person so that continuous direct observation is possible, or to a secure unit where available.
   - Involve family or other carers.
   - Consider one to one staffing

5. If the behaviour continues and is causing distress to the resident, staff and/or other residents call DBMAS for additional support

The DBMAS central number is 1800 699 799. This is a 24 hour number and the DBMAS team will then triage the call and allocate it to the best / first available person.

Assessment and Management of People with Behavioural and Psychological Symptoms of Dementia (BPSD) NSW Health (https://www.ranzcp.org/Files/Publications/A-Handbook-for-NSW-Health-Clinicians-BPSD_June13_W.aspx)

6. If all non-pharmacological strategies have failed and acute sedation is needed, the following should be used as a guide to immediately reduce risks to the person, other patients and staff in the immediate environment#
   - Remember the principles of communication, especially body language and tone of voice.
   - Remember that the person with dementia will be afraid and confused
   - Oral medication should always be offered before using parenteral treatment.
   - Intramuscular (IM) medication can aggravate the person's distress and should only be used if the person cannot be calmed with oral medication. Depot antipsychotics such as Acuphase, Clopixol, Modecate and Haldol should never be used in this context.
   - Recommended medication#:
     Refer to the resident's Deprescribing Protocol which will have emergency PRN medication listed. Recommended#:

Medication#		Initial dose	Maximum total per event	Caution
Benzodiazepine (preferred)	Oxazepam	7.5mg	Not greater than 30mg in 24 hours	**Respiratory depression, confusion, ataxia**

SUMMARY#

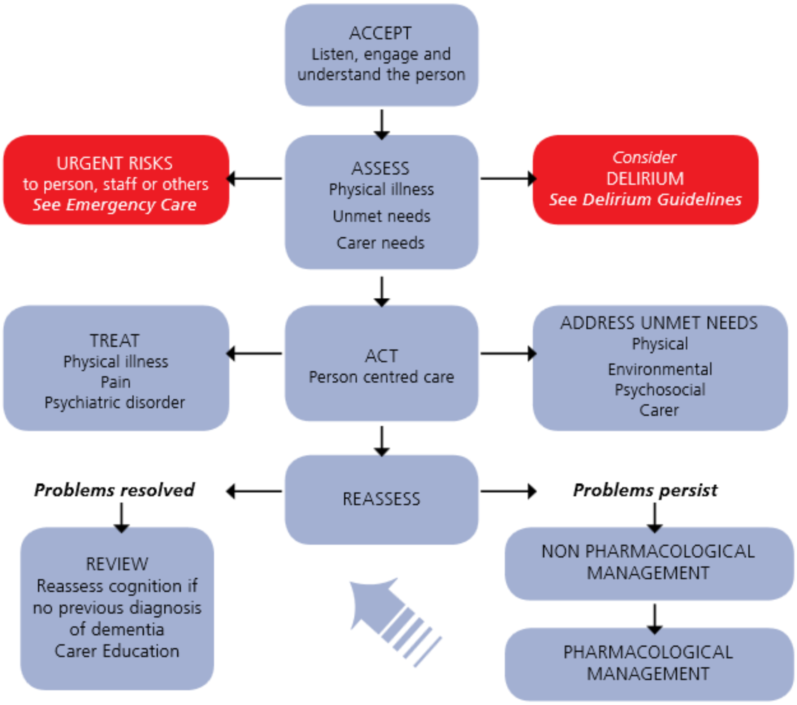

Any time that this protocol needs to be used, especially if PRN medication is administered, please contact GP (usual care) followed by HALT Coordinator Tiffany Jessop on 9385 2597 (office) 0414 754 894 (mobile) or t.holmes@unsw.edu.au as soon as practical.

Assessment and Management of People with Behavioural and Psychological Symptoms of Dementia (BPSD) NSW Health (https://www.ranzcp.org/Files/Publications/A-Handbook-for-NSW-Health-Clinicians-BPSD_June13_W.aspx)
